# Machine-Learning-Assisted Viscoelastic Characterization of PC/ABS Blends via Multi-Frequency Dynamic Mechanical Analysis

**DOI:** 10.3390/polym18050599

**Published:** 2026-02-28

**Authors:** Yancai Sun, Wenzhong Deng, Haoran Wang, Ranran Jian, Wenjuan Bai, Dianming Chu, Peiwu Hou, Yan He

**Affiliations:** 1 College of Electromechanical Engineering, Qingdao University of Science and Technology, Qingdao 266061, China; 2 Guangxi Key Laboratory of Special Engineering Equipment and Control, Guilin University of Aerospace Technology, Guilin 541004, China; 3 University Engineering Research Center of Non-Standard Intelligent Equipment and Process Control Technology, Guilin 541004, China; 4 Shandong Province Key Laboratory of Rubber-Based High-Performance Composites and Advanced Manufacturing, Qingdao 266061, China; 5 Design and Research Institute, China National Chemical Engineering Sixth Construction Co., Ltd., Wuhan 430074, China; 6 Department of Mechanical and Electrical Engineering, Qingdao University, Qingdao 266071, China

**Keywords:** PC/ABS blend, dynamic mechanical analysis, machine learning, physics-informed neural network, time–temperature superposition, viscoelasticity

## Abstract

This study combines multi-frequency dynamic mechanical analysis (DMA) with machine learning (ML) to characterize and predict the viscoelastic properties of a commercial polycarbonate/acrylonitrile–butadiene–styrene (PC/ABS) blend. DMA temperature sweeps at four frequencies (1–10 Hz) in single cantilever mode yielded a glass transition range of 115.8–123.2 °C ($E^{\prime \prime }$ peak), frequency sensitivity of 7.18 °C/decade, and an apparent activation energy of $335\pm 85\;kJ\;{\mathrm{mol}}^{-1}$. Time–temperature superposition master curves were parameterized with a six-term Prony series ($R^{2}=0.998$). Four data-driven models (RF, XGB, SVR, MLP) and a physics-informed NeuralWLF model were evaluated through a hierarchical validation framework. Temperature-blocked CV ranked MLP ($\bar{R^{2}}=0.989$) above RF (0.950) for interpolation; LOFO validation revealed that NeuralWLF achieved the best cross-frequency generalization ($R^{2}>0.92$ for all targets) with interpretable WLF parameters ($C_{1}\approx 12.2$, $C_{2}\approx 51.7\;{}^{\circ}C$). A systematic block size sweep (5–30 °C) revealed a validation inflation effect in which MLP tanδ$R^{2}$ dropped from 0.986 to 0.592 as the gap-to-FWHM ratio increased from 0.5 to 3.1, establishing the gap/FWHM ratio as a quantitative validation stringency criterion. A physics–data crossover was identified at gap/FWHM ≈2: beyond this threshold, NeuralWLF outperformed all data-driven models in tanδ prediction by up to +0.300 in $R^{2}$, while curriculum learning (freezing the WLF layer for 300 epochs) further improved the most stringent 30 °C validation from $R^{2}=0.660$ to 0.731. The integrated framework demonstrates that honest evaluation of DMA–ML models requires validation gaps exceeding the characteristic feature width and introduces a quantifiable physics-data crossover criterion for selecting between data-driven and physics-informed architectures.

## 1. Introduction

Polycarbonate/acrylonitrile–butadiene–styrene (PC/ABS) blends constitute one of the most commercially significant families of engineering thermoplastics, combining the impact resistance and heat distortion resistance of polycarbonate (PC) with the processability and cost advantages of ABS [1,2]. These blends are extensively deployed in automotive, electronics, and consumer product applications and have increasingly been adopted as feedstock materials for fused deposition modeling and other additive manufacturing processes [3,4]. Across these applications, the mechanical performance under service conditions is governed by the time- and temperature-dependent viscoelastic response, making accurate characterization across broad frequency and temperature domains essential for engineering design.

Dynamic mechanical analysis (DMA) is the principal experimental technique for measuring the viscoelastic properties of polymeric materials [5,6]. By subjecting a specimen to small-amplitude sinusoidal deformation at controlled frequency and temperature, DMA provides simultaneous determination of the storage modulus ($E^{\prime }$), loss modulus ($E^{\prime \prime }$), and loss tangent (tanδ). Multi-frequency DMA experiments enable systematic investigation of the frequency dependence of relaxation processes, yielding kinetic parameters such as the apparent activation energy for segmental motion [7,8]. The glass transition temperature ($T_{g}$), the key parameter governing the service performance of amorphous polymers, can be determined from the temperature at which $E^{\prime \prime }$ or tanδ reaches its maximum [9].

For polymer blends, the glass transition behavior is inherently more complex than for homopolymers. In immiscible systems such as PC/ABS, the blend typically exhibits distinct glass transitions corresponding to the PC-rich and ABS-rich phases [10,11]. The PC-phase $T_{g}$, which dictates the upper service temperature, is commonly observed in the range of 120–135 °C in blends, depressed from the 145–155 °C characteristic of neat PC [6,11]. The overlap of the PC- and SAN-phase (the matrix component of ABS) relaxations complicates both the extraction of $T_{g}$ and the application of analytical superposition methods.

Time–temperature superposition (TTS) exploits the equivalence between the effects of frequency and temperature on viscoelastic response to construct master curves spanning frequency ranges far exceeding those accessible in a single experiment [5,12]. For thermorheologically simple materials, the shift factors follow the Williams–Landel–Ferry (WLF) equation or an Arrhenius relationship near $T_{g}$ [13,14]. However, immiscible polymer blends are inherently thermorheologically complex: the distinct relaxation mechanisms in each phase possess different temperature dependencies, leading to imperfect superposition [15,16,17]. For PC/ABS systems, the degree of TTS failure depends on composition and morphology, with PC-rich blends exhibiting more satisfactory superposition due to the dominance of the PC-phase relaxation [11].

Once TTS master curves are obtained, the relaxation behavior can be parameterized through a Prony series representation, expressing the relaxation modulus as a sum of exponential decay terms suitable for direct implementation in finite element method (FEM) software [18,19,20]. For immiscible blends where TTS is imperfect, the resulting Prony parameters carry inherent uncertainties that are difficult to quantify within the traditional analytical framework.

Machine learning (ML) methods have demonstrated growing capability in predicting the properties of polymeric materials [21,22]. Supervised regression algorithms can capture complex nonlinear relationships between testing conditions and material properties without requiring explicit physical models. ML approaches have been applied to predict glass transition temperatures from molecular descriptors [23], to establish structure–property relationships for polymer composites [24], and to forecast mechanical performance under varying environmental conditions [25]. More broadly, recent materials–ML studies have expanded toward integrated discovery workflows, including surrogate modeling, inverse design, and data-driven exploration at scale [26,27]. Recent polymer-focused work has also moved toward physics-guided learning, including large-deformation viscoelastic prediction [28], physics-enforced neural modeling of polymer melt viscosity [29], and systematic reviews of physics-informed neural networks in polymer research [30]. These advances motivate the use of hybrid ML models for polymer mechanics, but they also show that model validity must be tied to well-defined experimental domains. In particular, direct learning of the DMA triplet $\left(E^{\prime },E^{\prime \prime },\tan\delta \right)$ over coupled temperature–frequency space with explicit cross-frequency extrapolation diagnostics remains limited.

ML-based prediction of DMA properties offers distinct advantages over the traditional TTS framework. First, ML models do not require the assumption of thermorheological simplicity, which is inherently violated in immiscible blends. Second, once trained, ML models provide instantaneous predictions at arbitrary temperature–frequency combinations. Third, feature importance metrics from tree-based methods offer quantitative insight into the relative contributions of temperature and frequency to the viscoelastic response. Nevertheless, the extrapolation capabilities of ML models beyond the training domain have not been systematically assessed.

In this study, we present an integrated DMA–ML framework for the viscoelastic characterization of a commercial PC/ABS blend. Multi-frequency DMA measurements in single cantilever mode are supplemented by dual cantilever geometry verification. Traditional analysis methods—frequency-dependent $T_{g}$ determination, Arrhenius activation energy, TTS master curves, and Prony series fitting—are applied alongside four data-driven ML regression models (RF, XGB, SVR, MLP) and a physics-informed NeuralWLF model that embeds a differentiable WLF layer within a neural network. A leave-one-frequency-out (LOFO) validation strategy is introduced to assess frequency extrapolation capability. Relative to prior polymer–ML studies, our emphasis is on experimentally grounded prediction of $\left(E^{\prime },E^{\prime \prime },\tan\delta \right)$ under standard DMA conditions and on making the in-domain/extrapolation boundary explicit. The objectives are threefold: (1) to establish a comprehensive DMA characterization with engineering-approximate constitutive parameters; (2) to evaluate interpolation and extrapolation performance of data-driven and physics-informed ML models; and (3) to compare ML insights with physical understanding from traditional analysis.

## 2. Materials and Methods

### 2.1. Materials

The material investigated was a commercial-grade PC/ABS blend. The supplier trade name and technical datasheet were not available for the specific grade; the nominal composition of approximately 40:60 PC:ABS by weight is based on supplier verbal communication and is used as a working assumption throughout this study. Accordingly, the material identity is characterized primarily by its measured DMA fingerprint ($E_{\mathrm{glassy}}^{\prime }\approx 1871\;\mathrm{MPa}$, $T_{g,E^{\prime \prime }}=115.8\;{}^{\circ}C$ at 1 Hz, $T_{g,\tan\delta }=123.5\;{}^{\circ}C$ at 1 Hz). No resolved secondary tanδ peak was observed in the DMA traces, suggesting either peak merging between the PC and SAN phases or a dominant single-phase response; additive content (fillers, flame retardants) cannot be inferred from DMA alone, and the detailed formulation is not disclosed.

Material characterization limitations. Complementary thermal analysis (DSC, TGA) and spectroscopic characterization (FTIR) were not performed in this study. Consequently, (i) the PC:ABS ratio remains unverified beyond nominal supplier information, (ii) the phase morphology (miscibility, domain size) is unknown, and (iii) the presence and concentration of additives (stabilizers, flame retardants, impact modifiers) cannot be assessed. The single observed $T_{g}$ in DMA could reflect either a miscible blend, overlapping phase transitions, or PC-dominated response; distinguishing among these scenarios would require DSC with controlled heating rates or dynamic rheology at lower frequencies. Readers seeking to reproduce or extend this work should treat the material as a “black-box” commercial formulation characterized by its DMA response rather than a compositionally defined system.

Test specimens were injection molded into rectangular bars of approximately 35×12×3 mm (L×W×T) for DMA testing. Prior to measurement, all specimens were dried at 80 °C for a minimum of 4 h to remove absorbed moisture.

### 2.2. DMA Measurements

Dynamic mechanical analysis was performed using a commercial dynamic mechanical analyzer operated in both single cantilever and dual cantilever bending modes. Temperature sweep experiments were conducted over the range of 30–170 °C at a heating rate of 3 °C min^−1^ under a nitrogen atmosphere.

Single cantilever mode. The primary characterization was performed in single cantilever geometry. Multi-frequency measurements were carried out simultaneously at five test frequencies: 0.5, 1, 2, 5, and 10 Hz. The oscillation amplitude was maintained within the linear viscoelastic region, confirmed by amplitude sweeps (5–50 μm) showing <5% variation in $E^{\prime }$. For the single cantilever geometry employed (free length ≈17.5 mm, thickness ≈3 mm), the applied amplitude of 15 μm corresponds to a maximum surface strain of approximately 0.015%, well within the typical linear viscoelastic limit for glassy polymers. Storage modulus ($E^{\prime }$), loss modulus ($E^{\prime \prime }$), and loss tangent (tanδ) were recorded as functions of temperature at each frequency. Approximately 930 data points were acquired per frequency, with the exception of the 0.5 Hz channel (94 points; see Section 3.2). The 1–10 Hz analysis window was selected for three practical reasons. First, it provided dense and stable sampling in the multi-frequency acquisition mode under a fixed thermal ramp. Second, it covered the main transition kinetics with acceptable signal quality across all retained frequencies. Third, this single-decade band is commonly used for comparative DMA characterization under engineering test conditions. We emphasize that model conclusions in this work are therefore validated within this band; extrapolation beyond 1–10 Hz should be treated as out-of-domain unless additional data are acquired.

Dual cantilever mode. To provide an independent geometry verification of the single cantilever results, three additional specimens (designated A, B, and C) were tested in dual cantilever mode at frequencies of 0.5, 1.0, and 2.0 Hz, respectively. The dual cantilever geometry constrains both specimen ends and applies a central deflection, producing a different stress distribution from the single cantilever configuration. Comparison of $T_{g}$ values and damping characteristics between geometries provides a quantitative robustness check (see Section 3.2).

### 2.3. Traditional Analysis Methods

Glass transition temperature. $T_{g}$ was determined by two standard methods: (i) the tanδ peak maximum ($T_{g,\tan\delta }$) and (ii) the $E^{\prime \prime }$ peak maximum ($T_{g,E^{\prime \prime }}$) [9].

Frequency dependence and activation energy. The frequency sensitivity was quantified as $dT_{g}/d\left(\log f\right)$ via linear regression. The apparent activation energy ($E_{a}$) was calculated from the Arrhenius equation:

(1) $$\ln f=\ln f_{0}-\frac{E_{a}}{R}\;\frac{1}{T_{g}}$$

where *R* = 8.314 J mol^−1^ K^−1^.

Time–temperature superposition. TTS master curves were constructed at $T_{\mathrm{ref}}=115.8\;{}^{\circ}C$. Shift factors ($a_{T}$) were determined empirically and analyzed using the Arrhenius formalism.

Prony series fitting. The storage modulus master curve was parameterized using a generalized Maxwell model:

(2) $$E^{\prime }\left(\omega \right)=E_{\infty }+\sum_{i=1}^{N}E_{i}\;\frac{{\left(\omega \tau_{i}\right)}^{2}}{1+{\left(\omega \tau_{i}\right)}^{2}}$$

where $E_{\infty }$ is the equilibrium modulus, $E_{i}$ and $\tau_{i}$ are the modulus and relaxation time of the *i*-th element, $\omega =2\pi f_{r}$ is the angular frequency corresponding to the reduced frequency $f_{r}=f\;\cdot \;a_{T}$, and N=6 terms were employed, optimized by nonlinear least-squares regression. Throughout this work, master curves and Prony fits are plotted against $f_{r}$ (Hz) rather than $\omega_{r}$ (rad/s) for consistency with the experimental frequency axis.

### 2.4. Machine Learning Methodology

Data preparation. DMA data from four reliable test frequencies (1, 2, 5, and 10 Hz) were compiled into a unified dataset of 3751 samples (1 Hz: 951; 2 Hz: 935; 5 Hz: 930; 10 Hz: 935). The 0.5 Hz data were excluded due to sparse sampling (94 points). No data points were removed during cleaning (no missing values, no outlier rejection); the count differences arise from minor variations in instrument sampling across frequencies. No interpolation or temperature-grid alignment was applied; each frequency retained its native temperature sampling. All four frequencies cover the full 30–170 °C range, ensuring that LOFO train/test partitions differ only in frequency, not in temperature coverage. Each sample comprised two input features—temperature (*T*) and $\log_{10}f$—and three targets: $\log_{10}E^{\prime }$, $\log_{10}E^{\prime \prime }$, and tanδ.

Model selection. Five regression models were evaluated:

(1) *Random forest (RF)*: bootstrap-aggregated decision tree ensemble [31];
(2) *Extreme gradient boosting (XGB)*: sequential ensemble with regularization [32];
(3) *Support vector regression (SVR)*: kernel-based hyperplane fitting [31];
(4) *Multilayer perceptron (MLP)*: feedforward neural network [31];
(5) *NeuralWLF* (physics-informed): a hybrid model embedding the WLF equation as a differentiable PyTorch layer, described below.

All data-driven models were implemented using standard hyperparameters selected a priori from preliminary experiments on a dedicated tuning block (the 70–80 °C glassy-region block, which was excluded from all 14 reported CV test folds to ensure strict separation between tuning and evaluation data); no per-fold hyperparameter re-optimization was performed. Input features were standardized (zero mean, unit variance), with the standardization parameters fitted exclusively on the training partition of each fold and applied to the corresponding test partition to prevent information leakage through preprocessing.

NeuralWLF architecture. The NeuralWLF model is a hybrid model embedding the WLF equation as a differentiable PyTorch layer. The architecture consists of (a) a differentiable WLF layer computing $\log a_{T}=-C_{1}\left(T-T_{\mathrm{ref}}\right)/\left(C_{2}+T-T_{\mathrm{ref}}\right)$; (b) a shared fully connected backbone receiving $\left(T,\log_{10}f,\log_{10}f_{r}\right)$; and (c) three output heads for $\log_{10}E^{\prime }$, $\log_{10}E^{\prime \prime }$, and tanδ. The backbone uses width 128 with GELU activations (num_layers = 4 in code, corresponding to three hidden transforms before the output heads). The tanδ head uses Softplus to enforce non-negative outputs. For CV/LOFO experiments, optimization used Adam with separate learning rates for neural parameters (5 × 10^−3^) and WLF parameters (1 × 10^−3^), warmup of 50 epochs, ReduceLROnPlateau scheduling (patience 100, factor 0.5), early stopping patience 600, and 5 random restarts per fold under deterministic seeds. Additional full-physics runs for K-K plausibility used $\lambda_{\mathrm{KK}}=0.02$, $\lambda_{\mathrm{mono}}=0.05$, and curriculum training with WLF freezing for the first 300 epochs [33,34]. Detailed network/training settings are summarized in Table 1.

Cross-validation strategy. Two complementary validation strategies were employed:

(i) *Temperature-blocked CV*: The temperature axis was partitioned into 10 °C blocks (14 blocks total). In each fold, all data within one block were held out as the test set, preventing data leakage from the densely sampled DMA data (autocorrelation r>0.999 at 0.15 °C spacing).

(ii) *Leave-one-frequency-out (LOFO) validation*: Each of the four training frequencies was held out in turn. LOFO assesses extrapolation capability—predicting properties at a frequency entirely absent from the training data.

Reproducibility and limitations. All stochastic operations (RF bootstrap, MLP weight initialization, permutation importance shuffling) used a fixed global random seed (RANDOM_STATE = 42). Temperature-blocked CV boundaries were generated deterministically as np.arange (30, 180, 10), yielding 14 blocks of 10 °C width. All models were trained from scratch within each CV or LOFO fold; no pre-trained weights were carried across folds. The entire dataset derives from a single injection-molded specimen per test condition; consequently, the results characterize measurement variability (instrument noise, within-specimen heterogeneity) but do not capture batch-to-batch or specimen-to-specimen variation. Cross-batch generalization should not be assumed without additional replication.

Performance metrics. $R^{2}$ and RMSE were calculated separately for each target variable. For the modulus targets, both metrics were computed in the $\log_{10}$ domain (i.e., RMSE is in units of log_10_ MPa); for tanδ, metrics were computed directly in the untransformed domain. A negative $R^{2}$ indicates that the model’s predictions are worse than a constant-mean baseline (i.e., the residual sum of squares exceeds the total sum of squares), as can occur when a model is evaluated on data outside its training distribution.

## 3. Results and Discussion

### 3.1. DMA Temperature Sweep Characteristics

**Figure 1:**
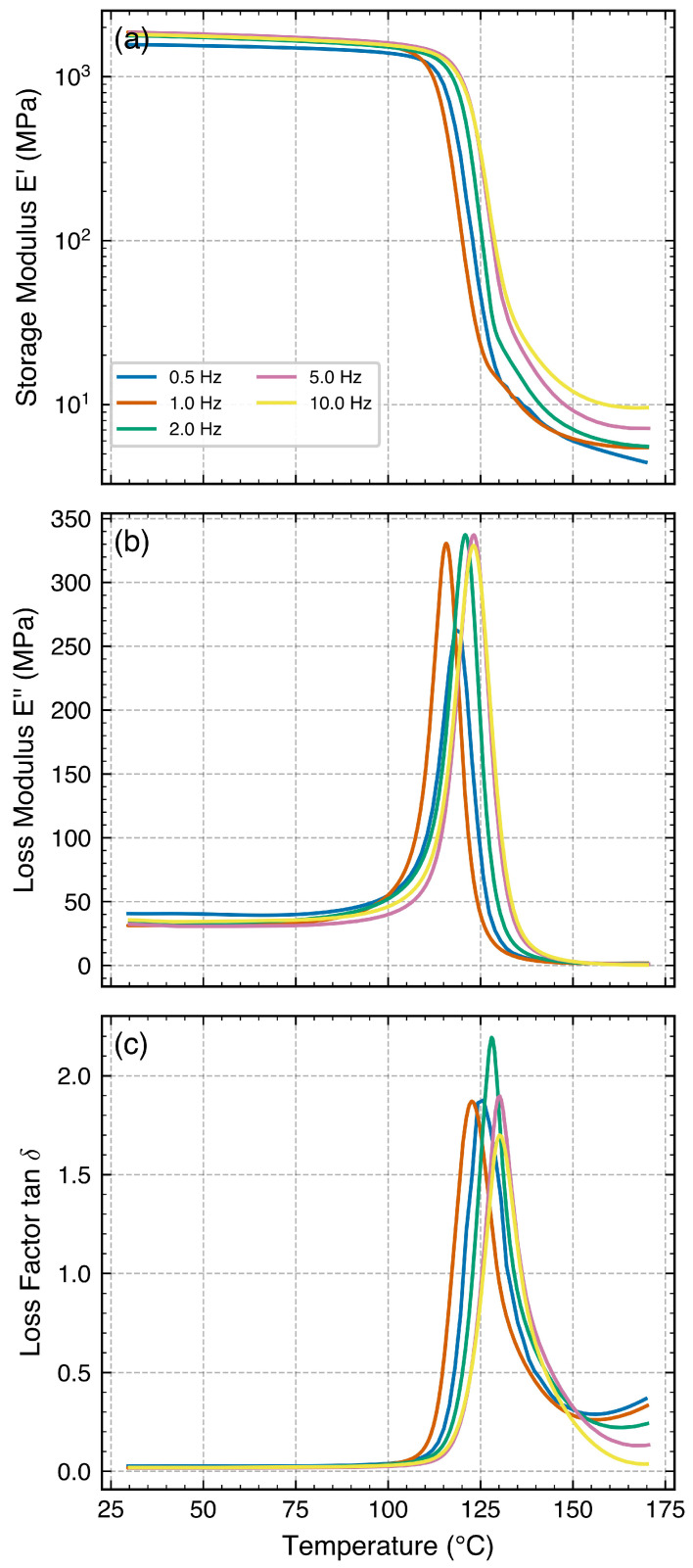
Temperature-dependent viscoelastic properties of the PC/ABS blend measured in single cantilever mode at five frequencies (0.5–10 Hz): (**a**) storage modulus $E^{\prime }$, (**b**) loss modulus $E^{\prime \prime }$, and (**c**) loss tangent tanδ. The systematic shift of the glass transition to higher temperatures with increasing frequency reflects the kinetic nature of the α-relaxation. All frequencies exhibit a glassy plateau near 1800 MPa and a rubbery plateau of 5–10 MPa. The 0.5 Hz data (94 points) show an anomalously sharp tanδ peak due to insufficient sampling density.

Figure 1 presents the storage modulus ($E^{\prime }$), loss modulus ($E^{\prime \prime }$), and loss tangent (tanδ) as functions of temperature at five frequencies. The storage modulus exhibited a glassy plateau of approximately 1871 MPa below 100 °C, followed by a steep decrease through the glass transition region to a rubbery plateau of approximately 5.5 MPa above 150 °C. The modulus decrease of more than two orders of magnitude is characteristic of cooperative segmental relaxation (α-relaxation).

The loss modulus displayed a well-defined peak at 115.8 °C (1 Hz), with a peak magnitude of 330 MPa. The tanδ peak occurred at 123.5 °C with a maximum value of 2.36 and a full width at half maximum (FWHM) of 9.7 °C. The 7.7 °C offset between the $E^{\prime \prime }$ and tanδ peak positions reflects the mathematical relationship $\tan\delta =E^{\prime \prime }/E^{\prime }$ [5,6].

No clearly resolved secondary tanδ peak attributable to the ABS phase was observed in the measured temperature range (30–170 °C). Visual inspection of the $E^{\prime \prime }$ traces in the 100–120 °C range revealed no discernible shoulder or inflection that would indicate a separate SAN-phase relaxation; however, peak deconvolution was not attempted, and a low-amplitude SAN contribution partially merged with the dominant PC peak cannot be excluded at the present signal-to-noise level and temperature resolution. The absence of a resolved secondary peak is consistent with—but does not uniquely prove—a scenario where the SAN-phase $T_{g}$ (typically 100–115 °C) overlaps with the onset of the PC relaxation [10,11]; confirming the phase structure would require complementary techniques such as DSC or dynamic rheology at lower frequencies. Table 2 summarizes the key DMA parameters.

### 3.2. Frequency Dependence and Activation Energy

**Figure 2:**
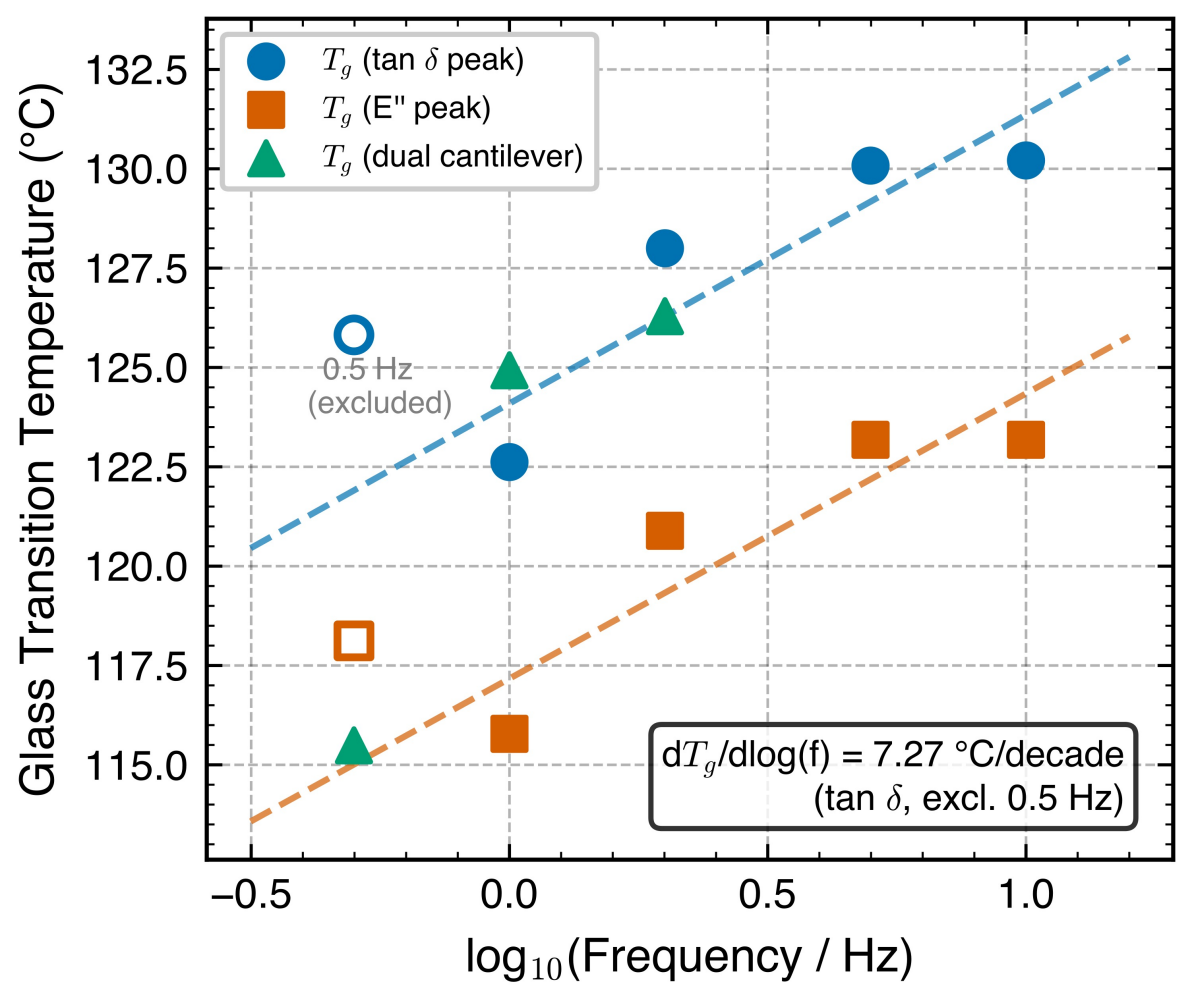
Glass transition temperature as a function of $\log_{10}$(frequency) determined by the tanδ peak (circles), $E^{\prime \prime }$ peak (squares), and dual cantilever measurements (triangles). Dashed lines represent linear fits. The 0.5 Hz point is labeled “excluded” due to insufficient data density. Dual cantilever $T_{g,\tan\delta }=123.1\;{}^{\circ}C$ at 1 Hz agrees with the single cantilever value (123.5 °C) to within 0.5 °C.

Both $T_{g,\tan\delta }$ and $T_{g,E^{\prime \prime }}$ shifted to higher temperatures with increasing frequency, consistent with the principle that higher frequencies probe shorter relaxation times [5,12]. Figure 2 presents $T_{g}$ versus $\log_{10}f$ with linear fits.

For the tanδ method (excluding the anomalous 0.5 Hz data), $dT_{g}/d\left(\log f\right)=6.69\;{}^{\circ}C/\mathrm{decade}$; the $E^{\prime \prime }$ method yielded 7.18 °C/decade (Table 3). The $E^{\prime \prime }$-based value of 7.18 °C/decade lies at the upper bound of the 3–7 °C/decade range commonly reported for PC/ABS blends [6,11,13]. This near-upper-bound value is compatible with a PC-dominated relaxation response typical of moderate PC fractions, though it may also reflect the limited frequency window (1–10 Hz, i.e., 4 data points for the regression), which amplifies sensitivity to individual $T_{g}$ determinations. No quantitative inference about the PC:ABS ratio can be drawn from the frequency sensitivity alone. The tanδ-based value of 6.69 °C/decade falls more centrally within the reported range.

**Figure 3:**
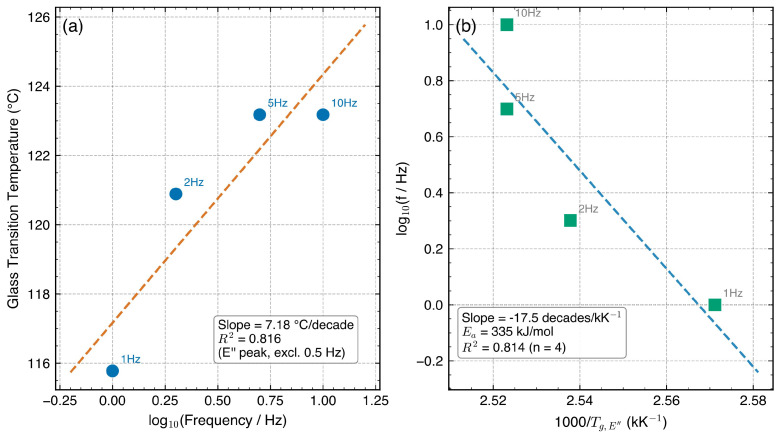
Frequency–temperature shift analysis: (**a**) $T_{g,E^{\prime \prime }}$ versus $\log_{10}$(frequency), with blue circles indicating measured data points and linear regression excluding 0.5 Hz; (**b**) Arrhenius plot of $\log_{10}\left(f\right)$ versus $1000/T_{g,E^{\prime \prime }}$ (kK^−1^) for determination of the apparent activation energy ($E_{a}=335kJ\;{\mathrm{mol}}^{-1}$).

The apparent activation energy was estimated from the Arrhenius analysis of the $E^{\prime \prime }$ peak shift (Figure 3b). Linear regression of $\log_{10}f$ versus $1000/T_{g,E^{\prime \prime }}$ yielded a slope of −17.5 $\log_{10}\left(\mathrm{Hz}\right)/\left({\mathrm{kK}}^{-1}\right)$ ($R^{2}=0.814$, n=4 frequencies), where the abscissa has units of $10^{3}\;K^{-1}$. Converting to activation energy via $E_{a}=-\mathrm{slope}\times 10^{3}\times 2.303\times R$: $E_{a}=17.5\times 10^{3}\times 2.303\times 8.314\times 10^{-3}\approx 335\;kJ\;{\mathrm{mol}}^{-1}$, with a 95% confidence interval of $\pm 85\;kJ\;{\mathrm{mol}}^{-1}$ from the regression slope uncertainty (Table 4). A leave-one-point-out sensitivity check on the 4-point regression yielded $E_{a}$ estimates of 261, 339, and 360 kJ mol^−1^ when the 10, 2, and 5 Hz points were individually omitted; however, omitting 1 Hz produced an outlier of 713 kJ mol^−1^ because the remaining three $T_{g}$ values span only 2.3 °C, making the regression ill-conditioned. This confirms that the 4-point Arrhenius estimate is order-of-magnitude stable (261–360 kJ mol^−1^ for the three well-conditioned subsets) but should be interpreted as an approximate value rather than a precisely determined quantity. The central estimate of 335 kJ mol^−1^ falls within the 200–400 kJ mol^−1^ range commonly reported for PC/ABS systems [11,13], consistent with PC-dominated cooperative segmental relaxation.

Anomalous 0.5 Hz data. The 0.5 Hz channel contained only 94 data points compared with ∼930 at each of the other four frequencies. This order-of-magnitude difference is attributed to the instrument’s multi-frequency acquisition mode, in which the lowest-frequency channel is recorded at a decimated rate to accommodate the longer oscillation period; upon data export, only one point per approximately 1.5 °C was retained (versus ∼0.15 °C at higher frequencies). The sparse sampling produced an anomalously sharp tanδ peak (4.22 versus 2.36 at 1 Hz) and narrow FWHM (4.7 °C versus 9.7 °C); the corresponding $T_{g,\tan\delta }$ of 125.8 °C violates the expected monotonic frequency–$T_{g}$ relationship. The $E^{\prime }$ glassy plateau at 0.5 Hz (1565 MPa, Table 2) is also ∼16% below the ∼1800 MPa level observed at 1–10 Hz; this depression is consistent with the glassy plateau not being fully resolved by the sparse temperature grid rather than a genuine material difference, because the overall $E^{\prime }\left(T\right)$ curve shape is consistent across all frequencies. These data were excluded from all quantitative analyses and ML training.

Geometry robustness check with dual cantilever mode. Independent specimens tested in dual cantilever mode at 1 Hz (specimen B) yielded $T_{g,\tan\delta }=123.1\;{}^{\circ}C$ and $T_{g,E^{\prime \prime }}=115.4\;{}^{\circ}C$, compared with 123.5 °C and 115.8 °C from single cantilever (Δ=0.4 °C for both definitions). The tanδ peak value was 2.43 (dual) versus 2.36 (single), a difference of approximately 3%. At 2 Hz (specimen C), $T_{g,\tan\delta }=125.4$ versus 128.0 °C (Δ=2.6 °C) and $T_{g,E^{\prime \prime }}=117.4$ versus 120.9 °C (Δ=3.5 °C). The larger discrepancy at 2 Hz may reflect specimen-to-specimen variability or the different stress distributions between the two clamping geometries. Overall, the close agreement at 1 Hz confirms that the extracted $T_{g}$ values are robust with respect to measurement geometry, while the 2 Hz discrepancy underscores that dual cantilever data serve as an independent geometry verification rather than a strict cross-validation.

### 3.3. TTS Master Curves and Prony Series Fitting

**Figure 4:**
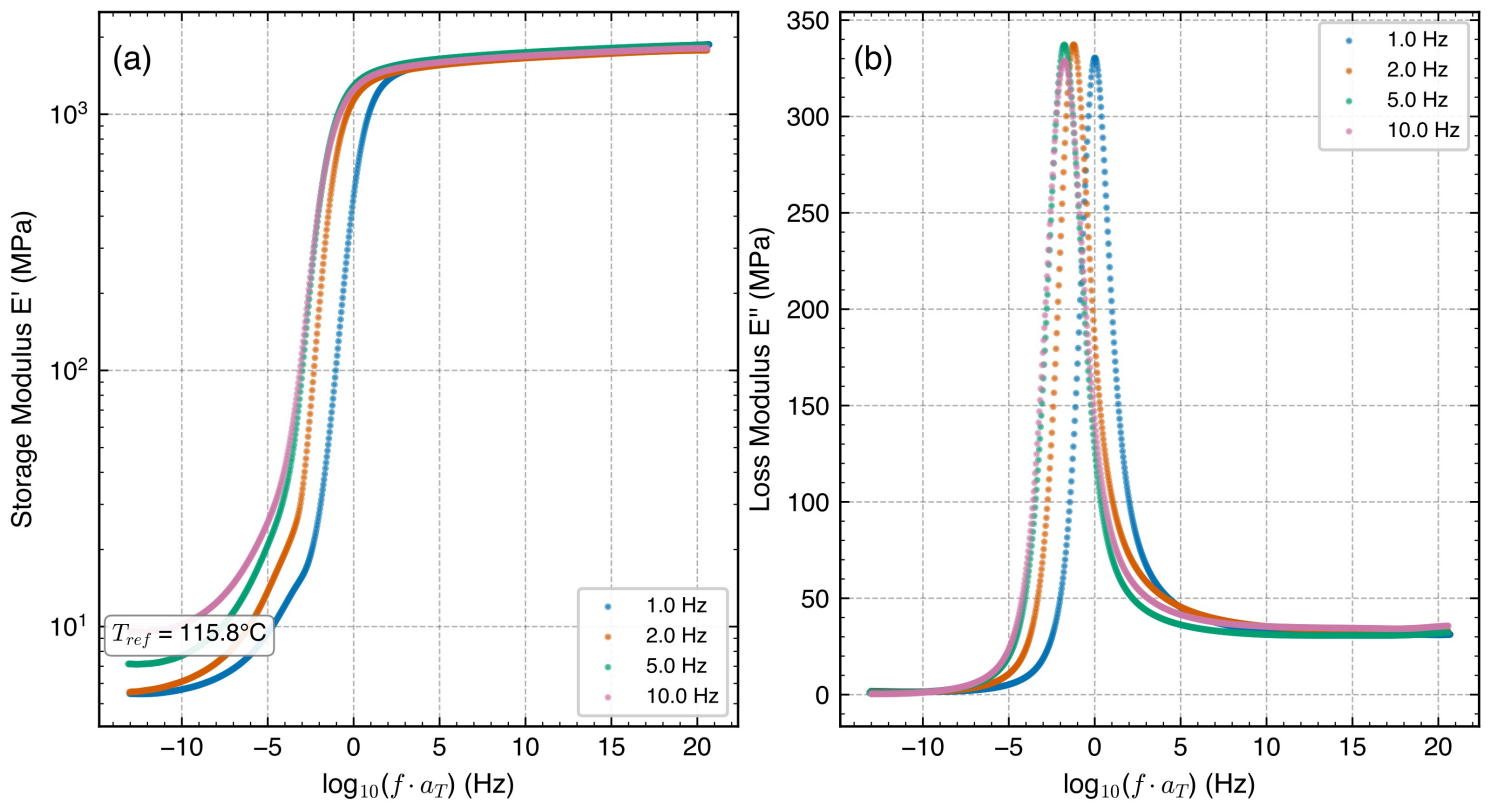
Time–temperature superposition master curves at $T_{\mathrm{ref}}=115.8\;{}^{\circ}C$: (**a**) storage modulus $E^{\prime }$ and (**b**) loss modulus $E^{\prime \prime }$ versus reduced frequency. Data from four frequencies (1, 2, 5, and 10 Hz) are superposed. The $E^{\prime }$ master curve exhibits satisfactory superposition spanning five frequency decades; $E^{\prime \prime }$ shows minor scatter from thermorheological complexity.

Master curves were constructed at $T_{\mathrm{ref}}=115.8\;{}^{\circ}C$ (Figure 4). The $E^{\prime }$ master curve spans approximately five decades of reduced frequency, from the rubbery plateau (∼5–10 MPa) to the glassy plateau (∼1800 MPa). The glassy-to-rubbery modulus ratio of ∼340 is comparable to reported values for PC/ABS blends [6,11]. Minor superposition imperfections in $E^{\prime \prime }$ reflect the thermorheological complexity of the immiscible blend [15,16].

**Figure 5:**
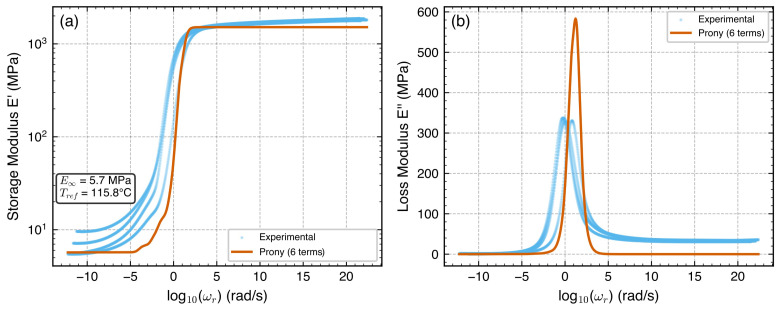
Prony series fit to the master curves: (**a**) storage modulus $E^{\prime }$ ($R^{2}=0.998$); (**b**) loss modulus $E^{\prime \prime }$. Both panels plotted against reduced frequency $f_{r}=f\;\cdot \;a_{T}$ (Hz) at $T_{\mathrm{ref}}=115.8\;{}^{\circ}C$. The Prony model uses $\omega =2\pi f_{r}$ internally. The $E^{\prime }$ fit is excellent across the full frequency range, while the $E^{\prime \prime }$ fit captures the primary peak but underestimates the experimental data in the transition region.

A six-term Prony series achieved $R^{2}=0.998$ (Table 5, Figure 5). The equilibrium modulus $E_{\infty }=5.4\;\mathrm{MPa}$ is consistent with the measured rubbery plateau. The dominant terms $E_{3}$–$E_{5}$ (τ=0.065–0.450 s) capture the primary glass transition and correspond to time scales observable within the experimental frequency band (f=1–10 Hz, equivalent to τ≈0.016–0.16 s). Term $E_{6}$ ($\tau =1.87\times 10^{-3}$ s) represents the fast glassy response; its relaxation time falls below the observable window and should be regarded as a weakly identifiable, extrapolative term that captures the steep high-frequency rise rather than a uniquely determined relaxation process. Terms $E_{1}$ and $E_{2}$, with relaxation times of $6.0\times 10^{6}$ and 3777 s, lie far outside the observable frequency window and similarly serve as mathematical baseline-correction terms that stabilize the fit at the low-frequency (rubbery) limit; their individual values are not physically identifiable from the present data and should not be interpreted as discrete relaxation processes. Restricting the fit to $\tau \in [10^{-3},10^{3}]$ s (N = 4 terms) yielded $R^{2}=0.994$, confirming that the observable-range terms capture the essential response with only marginal loss of accuracy. Notably, $E_{4}$ and $E_{5}$ share nearly identical relaxation times (∼0.065 s), indicating practical non-identifiability; a merged single term ($E_{4+5}\approx 869\;\mathrm{MPa}$, τ≈0.065 s) provides an equivalent representation. The Prony parameters in Table 5 should be regarded as engineering-equivalent fits valid at $T_{\mathrm{ref}}=115.8\;{}^{\circ}C$ within the experimental frequency band, rather than uniquely identifiable material constants. For FEM applications, these parameters should be used only within the calibrated reduced-frequency range ($f_{r}\approx 10^{-2}$–$10^{3}$ Hz); extrapolation beyond this range inherits the TTS imperfections of the immiscible blend and the non-identifiability of the boundary terms ($E_{1}$, $E_{2}$, $E_{6}$).

### 3.4. Damping Analysis

**Figure 6:**
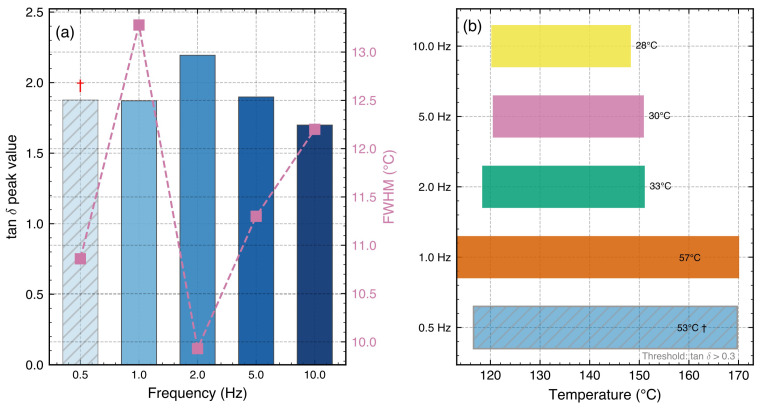
Damping performance characterization: (**a**) tanδ peak value (bars, left axis) and FWHM (squares, right axis) versus test frequency, showing inverse trends; (**b**) effective damping range (tanδ>0.3) at each frequency with temperature span indicated. The 0.5 Hz data (hatched bars, †) are shown for reference only and are excluded from quantitative trend analysis due to insufficient data density (94 points).

Figure 6 presents the damping characteristics for the four reliable frequencies (1–10 Hz). The tanδ peak height decreased systematically from 2.36 (1 Hz) to 1.70 (10 Hz), while the FWHM broadened from 9.7 °C to 12.2 °C. At higher frequencies, polymer chains have less time to respond within each cycle, resulting in broader, lower-amplitude transitions [5,7]. The effective damping range (tanδ>0.3, a pragmatic engineering threshold commonly used in vibration damping literature [5]) narrows from ∼57 °C at 1 Hz to ∼28 °C at 10 Hz; the trend is robust to modest threshold variations (e.g., 0.2–0.5) (The 0.5 Hz damping range (∼52 °C) is shown in Figure 6 for visual reference but is excluded from trend discussion due to sparse sampling; see Section 3.2.).

The preceding sections established the traditional DMA characterization of the PC/ABS blend: temperature sweep behavior across five frequencies, frequency-dependent $T_{g}$ determination with Arrhenius activation energy, time–temperature superposition master curves with Prony series parameterization, and damping performance characterization. While these analytical methods provide engineering-ready constitutive parameters, they rely on assumptions—most critically thermorheological simplicity—that may not strictly hold for immiscible blends such as PC/ABS. The following sections evaluate data-driven and physics-informed ML approaches as complementary tools for viscoelastic property prediction and assess their interpolation, extrapolation, and generalization capabilities.

### 3.5. ML Model Comparison: Temperature-Blocked Cross-Validation

**Figure 7:**
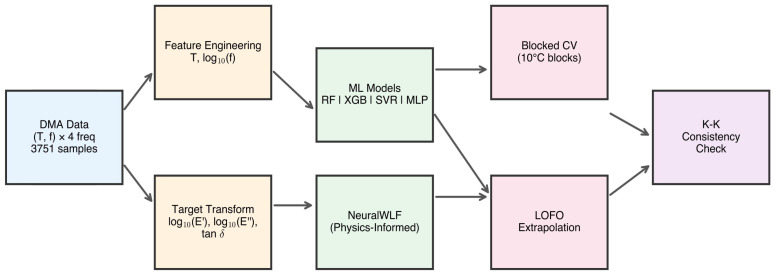
Machine learning workflow for viscoelastic property prediction. DMA data (3751 samples from 4 frequencies) undergo feature engineering (*T*, $\log_{10}f$) and target transformation ($\log_{10}E^{\prime }$, $\log_{10}E^{\prime \prime }$, tanδ). Four data-driven models (RF, XGB, SVR, MLP) and a physics-informed NeuralWLF model are evaluated through temperature-blocked CV (10 °C blocks) and LOFO validation.

The four data-driven ML models and NeuralWLF were evaluated using temperature-blocked CV (Figure 7). Table 6 summarizes the results.

**Figure 8:**
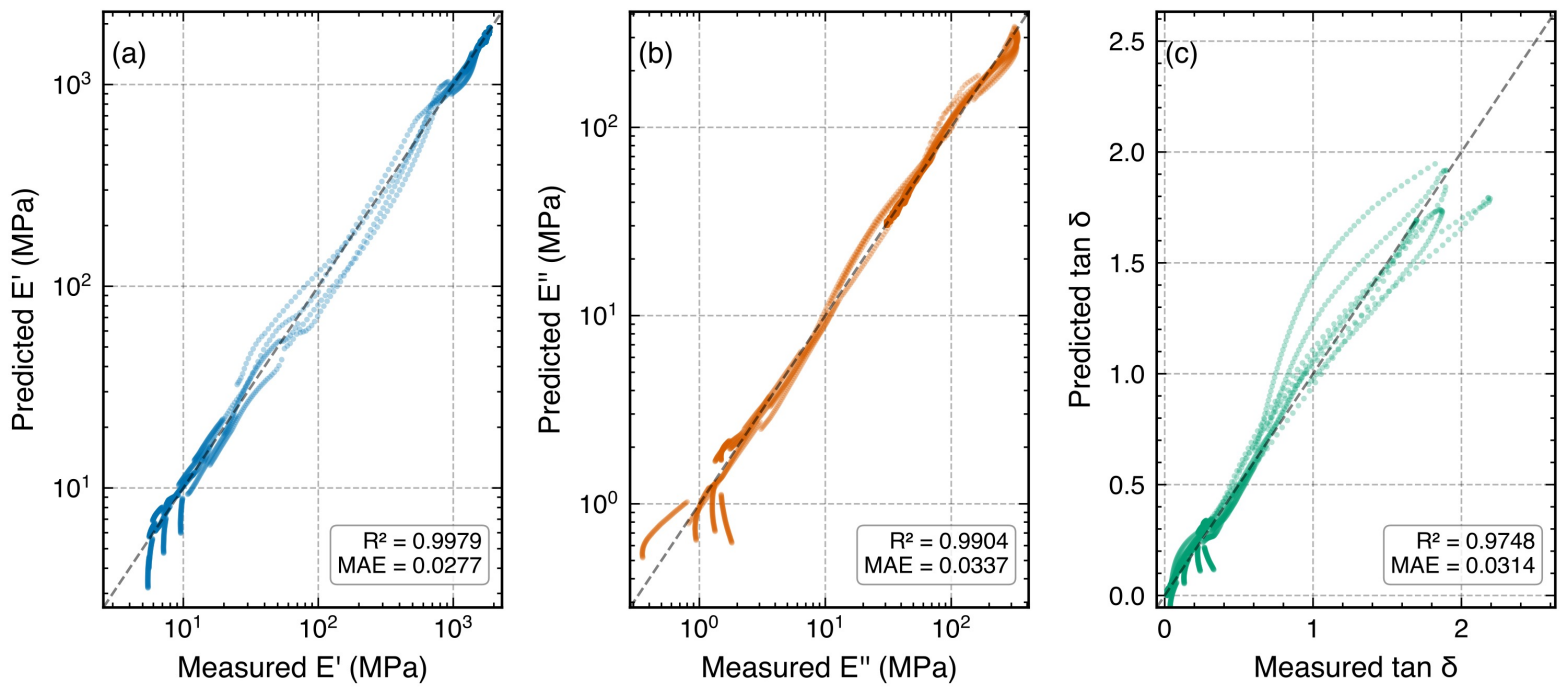
Parity plots of MLP-predicted versus measured values under temperature-blocked CV for: (**a**) $\log_{10}E^{\prime }$ ($R^{2}=0.997$), (**b**) $\log_{10}E^{\prime \prime }$ ($R^{2}=0.991$), and (**c**) tanδ ($R^{2}=0.978$). Points closely follow the y=x diagonal, with residual scatter concentrated in the steep glass transition region.

MLP achieved the highest $\bar{R^{2}}=0.989$, followed by RF (0.950). Both models captured the steep modulus transition through $T_{g}$ with high accuracy. MLP outperformed RF for all targets, with the largest advantage observed for tanδ ($R^{2}=0.978$ versus 0.889), suggesting that continuous function approximation is better suited than piecewise-constant tree-based representation for reproducing the smooth but steep damping peak. XGB ($\bar{R^{2}}=0.810$) and SVR (0.731) showed markedly lower accuracy, particularly for tanδ. Figure 8 presents the MLP parity plots. The physics-informed NeuralWLF is evaluated primarily via LOFO in Section 3.8. For comparability, NeuralWLF was also subjected to blocked CV restricted to the transition zone (90–150 °C, 6 of 14 blocks), where target variance is sufficient for meaningful $R^{2}$. Within this restricted range, NeuralWLF achieved $R^{2}=0.998$ ($\log_{10}E^{\prime }$), 0.998 ($\log_{10}E^{\prime \prime }$), and 0.985 (tanδ)—comparable to MLP. However, the full-range (all 14 blocks) MAE was 0.21 ($\log_{10}E^{\prime }$), 0.04 ($\log_{10}E^{\prime \prime }$), and 0.11 (tanδ), substantially exceeding MLP’s 0.03, 0.04, and 0.04, respectively. To enable direct comparison within the same temperature zone, the transition-zone RMSE (100–150 °C blocks only) was computed: NeuralWLF achieved 0.029/0.040/0.065 and MLP achieved 0.048/0.055/0.058 for $\log_{10}E^{\prime }$/$\log_{10}E^{\prime \prime }$/tanδ, respectively. Within the transition zone, the two models perform comparably; the full-range gap confirms that the WLF layer provides excellent frequency–temperature coupling within the transition zone but offers no advantage in the thermally flat plateau regions. The NeuralWLF model’s primary design goal—cross-frequency generalization—is assessed via LOFO in Section 3.8; its performance advantage is contingent on the narrow 1–10 Hz window where the WLF constraint is maximally informative.

The adoption of temperature-blocked CV is essential for reliable performance assessment. Preliminary experiments with conventional 5-fold CV yielded $R^{2}>0.999$, indicative of data leakage arising from the densely sampled data (r>0.999 between adjacent 0.15 °C-spaced points).

### 3.6. Frequency Extrapolation via LOFO Validation

**Figure 9:**
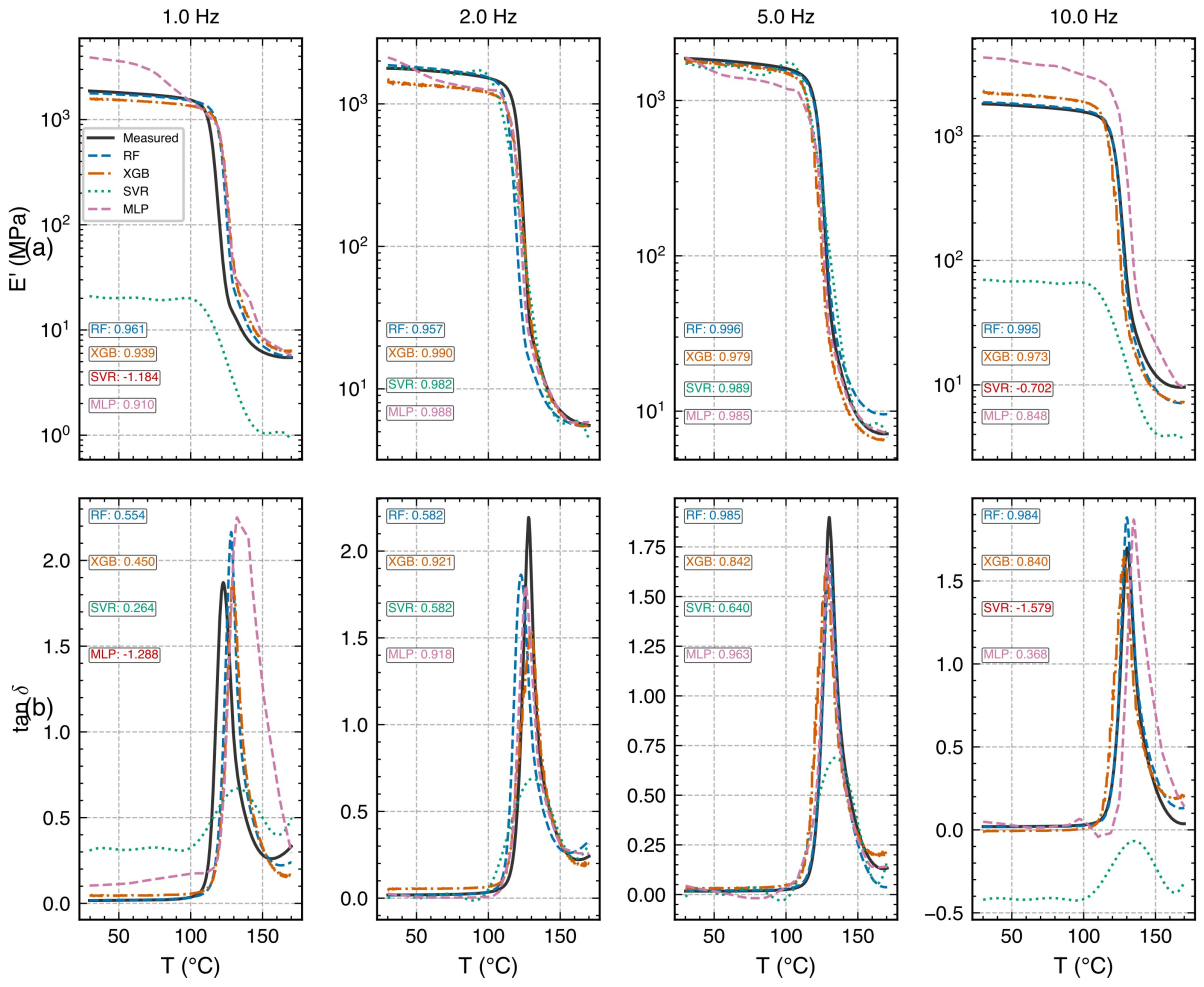
LOFO validation: predicted versus measured temperature sweeps at each held-out frequency for all four data-driven models (RF, XGB, SVR, MLP). Columns: 1, 2, 5, and 10 Hz; rows: (**a**) $E^{\prime }$ and (**b**) tanδ. Negative $R^{2}$ values (red) indicate model failure at edge frequencies. RF maintains positive $R^{2}$ throughout; SVR and MLP exhibit catastrophic failure at edge frequencies.

**Figure 10:**
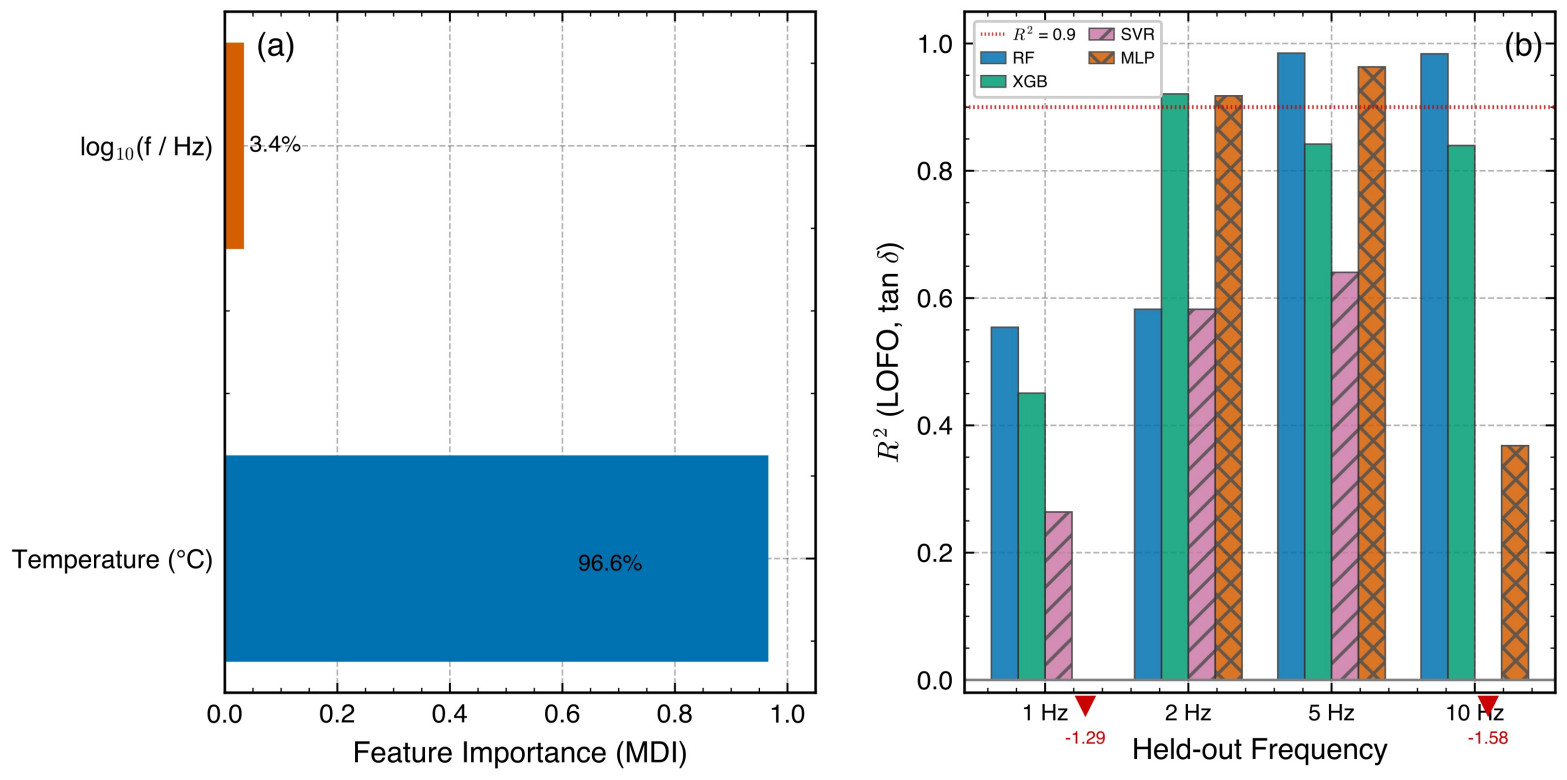
LOFO (**a**) Feature-importance ranking (RF, MDI) for tanδ prediction, showing the relative contributions of temperature and $\log_{10}\left(f\right)$; (**b**) tanδ$R^{2}$ comparison among all four data-driven models (RF, XGB, SVR, MLP) and the physics-informed NeuralWLF model at each held-out frequency. NeuralWLF maintains $R^{2}>0.92$ at all frequencies; RF and XGB maintain positive $R^{2}$ throughout; MLP collapses to $R^{2}=-0.97$ at 1 Hz; SVR fails catastrophically at both edge frequencies.

All four data-driven models and the NeuralWLF model were evaluated under LOFO to compare extrapolation behavior across model architectures. In the following discussion, “edge frequencies” refer to those at the boundaries of the training input space in $\log_{10}f$: 1 Hz ($\log_{10}f=0$, the low-frequency boundary) and 10 Hz ($\log_{10}f=1$, the high-frequency boundary); “interior frequencies” (2 and 5 Hz) lie within the convex hull of the training $\log_{10}f$ values. Predicting at an edge frequency requires extrapolation beyond the training support, whereas predicting at an interior frequency is interpolation. Results are presented in Table 7 and Figure 9 and Figure 10.

RF extrapolation. When interior frequencies (5, 10 Hz) are held out, RF predicts $E^{\prime }$ with $R^{2}>0.99$ and tanδ with $R^{2}>0.98$. At lower-edge frequencies (1, 2 Hz), $E^{\prime }$ remains strong ($R^{2}>0.95$) but tanδ degrades ($R^{2}=0.55$ and 0.49). Critically, RF maintains positive $R^{2}$ at all held-out frequencies and achieves the highest mean tanδ$R^{2}$ (0.75) among the four data-driven models.

XGB extrapolation. XGB exhibits a similar pattern to RF but with generally lower accuracy. At interior frequencies, XGB achieves tanδ$R^{2}=0.81$–0.92, comparable to RF. At the 1 Hz edge, tanδ$R^{2}=0.43$ (versus RF 0.55); at 10 Hz, tanδ$R^{2}=0.80$ (versus RF 0.98). The sequential boosting strategy provides moderate extrapolation robustness (mean tanδ$R^{2}=0.74$), slightly below RF, but does not exhibit the catastrophic failures observed in MLP and SVR at edge frequencies.

SVR extrapolation. SVR exhibited the worst extrapolation behavior among all models, with catastrophic failure at both edge frequencies: $E^{\prime }$$R^{2}=-1.18$ at 1 Hz and −0.70 at 10 Hz; tanδ$R^{2}=0.25$ at 1 Hz and −1.33 at 10 Hz. Even at interior frequencies, SVR underperformed (tanδ$R^{2}=0.58$–0.64). The kernel-based extrapolation is fundamentally limited by the decay of the RBF kernel outside the training support, which causes predictions to collapse toward the training-set mean.

MLP extrapolation. MLP achieves excellent interpolation at 2 and 5 Hz (tanδ$R^{2}=0.90$ and 0.97) but exhibits marked degradation at edge frequencies: tanδ$R^{2}=-0.97$ at 1 Hz and 0.38 at 10 Hz. In this implementation (fixed architecture, no monotonic constraints), MLP exhibits edge-frequency instability characteristic of neural networks operating outside the convex hull of training inputs.

NeuralWLF extrapolation. The physics-informed NeuralWLF achieved $R^{2}>0.92$ for all targets at all held-out frequencies (Table 7; Figure 10), including the challenging 1 Hz edge case where MLP collapsed to $R^{2}=-0.97$ and SVR to $R^{2}=-1.18$ for $E^{\prime }$. This robustness is attributable to the embedded WLF shift layer, which constrains the frequency dependence to follow a physically motivated functional form. The advantage is most pronounced at edge frequencies, where all four data-driven models exhibit degradation. However, this benefit is contingent on the validity of the WLF assumption within the tested frequency window; broader ranges or non-thermorheologically simple regimes could erode the physics-informed advantage.

Interpolation versus extrapolation tradeoff. Among purely data-driven models, MLP is the superior interpolator (blocked CV $\bar{R^{2}}=0.989$) but the second-worst extrapolator (mean LOFO tanδ$R^{2}=0.32$). RF provides the best extrapolation–interpolation balance (${\bar{R^{2}}}_{\mathrm{CV}}=0.950$, mean LOFO tanδ$R^{2}=0.75$). XGB offers comparable extrapolation to RF (0.74) with lower interpolation accuracy (0.810). SVR is unsuitable for frequency extrapolation (mean LOFO tanδ$R^{2}=0.04$). NeuralWLF achieves the best cross-frequency generalization ($R^{2}>0.92$ everywhere) at the cost of reduced interpolation in thermally flat regions (Section 3.5). The complementarity suggests that model selection should be guided by the prediction task: MLP for within-domain interpolation, RF or XGB for edge-frequency prediction, and NeuralWLF when physically interpretable frequency extrapolation is the primary objective.

Directional asymmetry and physical origin. All four data-driven models exhibit stronger performance at the high-frequency edge (10 Hz) than at the low-frequency edge (1 Hz). For RF, tanδ$R^{2}=0.98$ at 10 Hz versus 0.55 at 1 Hz; for XGB, 0.80 versus 0.43. This asymmetry has a physical origin: at lower frequencies, polymer chains have more time to relax within each cycle, producing a sharper, taller tanδ peak (2.36 at 1 Hz versus 1.70 at 10 Hz) with narrower FWHM (9.7 °C versus 12.2 °C). The sharper peak concentrates the signal in a narrower temperature window, making precise prediction more sensitive to small temperature offsets—an inherently harder extrapolation target than the broader, lower-amplitude peak at 10 Hz.

### 3.7. Feature Importance and Physical Interpretation

The mean decrease in impurity (MDI) feature importance for the RF model trained on the full dataset showed that temperature accounted for 96.6% of the total MDI, while $\log_{10}f$ contributed 3.4%. This pronounced dominance is partly physical—temperature sweeps spanning $T_{g}$ drive modulus changes exceeding two orders of magnitude, whereas the 1–10 Hz frequency range induces only a ∼7 °C shift per decade—but also partly artifactual: MDI is known to be biased toward high-cardinality continuous features [35], and temperature (∼940 unique values) offers far more split opportunities than $\log_{10}f$ (4 discrete levels).

As a robustness check, permutation importance (10 repeats per fold, computed on each blocked-CV test fold and size-weighted averaged) yielded an inverted ranking: temperature 26.3%, $\log_{10}f$ 73.7%. This reversal is explained by the blocked-CV design: within each 10 °C test block, temperature varies over a narrow range, whereas frequency spans all four levels, so shuffling frequency destroys most of the predictive signal in that context. Neither metric alone captures the complete picture; their disagreement exposes the known MDI cardinality bias while also reflecting the restricted within-block temperature variance of the blocked-CV evaluation. Taken together, both features are necessary: temperature drives the dominant physical variation across the glass transition, while frequency encodes the complementary time–temperature equivalence exploited by TTS analysis. The importance partition is conditional on the tested frequency window (1–10 Hz); a broader range (e.g., 0.01–100 Hz) would likely shift the balance.

### 3.8. NeuralWLF and Approximate Kramers–Kronig Plausibility

The NeuralWLF model is evaluated via LOFO rather than temperature-blocked CV. Per-fold neural-network training is ill-conditioned in the glassy plateau region where $E^{\prime }$ is nearly constant ($\log_{10}E^{\prime }$ standard deviation <0.02) and $R^{2}$ becomes undefined. LOFO, which holds out an entire frequency (25% of data), therefore provides a more meaningful assessment of cross-frequency generalization. In each LOFO fold, the NeuralWLF model—including all neural network weights and the learnable WLF parameters ($C_{1}$, $C_{2}$, $T_{\mathrm{ref}}$)—is trained from scratch using only the three non-held-out frequencies. The held-out frequency participates in neither parameter optimization nor hyperparameter selection, ensuring strict anti-leakage separation.

**Figure 11:**
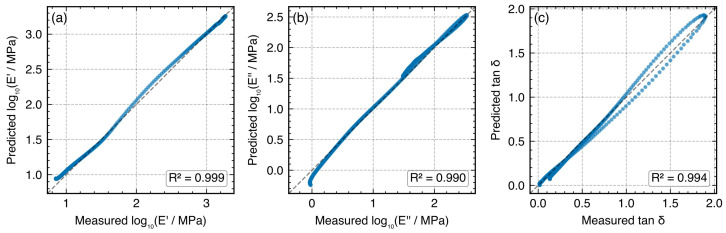
NeuralWLF parity plots for LOFO validation (held-out 5 Hz): (**a**) $\log_{10}E^{\prime }$ ($R^{2}=0.999$), (**b**) $\log_{10}E^{\prime \prime }$ ($R^{2}=0.990$), and (**c**) tanδ ($R^{2}=0.997$). The physics-informed model achieves strong cross-frequency generalization while providing interpretable WLF parameters.

Figure 11 presents LOFO parity plots for the held-out 5 Hz frequency (representative example). Across all four held-out frequencies, NeuralWLF achieved $R^{2}>0.96$ for both $E^{\prime }$ and $E^{\prime \prime }$ and $R^{2}>0.92$ for tanδ (Table 7). The lowest performance occurred at the edge frequency (1 Hz), where tanδ$R^{2}=0.922$—a known challenge for frequency extrapolation. The learned WLF parameters were consistent across LOFO folds ($C_{1}=12.14$–12.20, $C_{2}=51.68$–51.79 °C), confirming robust physical parameter recovery. Notably, the NeuralWLF model outperformed both RF and MLP on LOFO for edge frequencies, suggesting that physics-informed constraints provide a distinct advantage for frequency extrapolation that compensates for its limitations in temperature generalization.

Empirical uncertainty across held-out frequencies. To make the uncertainty level explicit, we summarize LOFO variability across the four held-out frequencies (computed from Table 7). NeuralWLF achieved mean tanδ performance of $R^{2}=0.974\pm 0.030$ (mean ± standard deviation), while MLP showed much larger spread ($R^{2}=0.320\pm 0.780$) because of edge-frequency collapse. For NeuralWLF, the corresponding means were $R^{2}=0.997\pm 0.003$ for $\log_{10}E^{\prime }$ and 0.982±0.012 for $\log_{10}E^{\prime \prime }$. This fold-to-fold dispersion provides an empirical uncertainty envelope for cross-frequency deployment within the tested 1–10 Hz window.

Approximate Kramers–Kronig plausibility check. Important caveat: Rigorous Kramers–Kronig validation requires master-curve data spanning multiple frequency decades; the single-decade bandwidth of the present study (1–10 Hz) is fundamentally insufficient for quantitative K-K compliance assessment. The following analysis is presented as a qualitative plausibility check of $E^{\prime }$–$E^{\prime \prime }$ covariation, not as thermodynamic validation.

**Figure 12:**
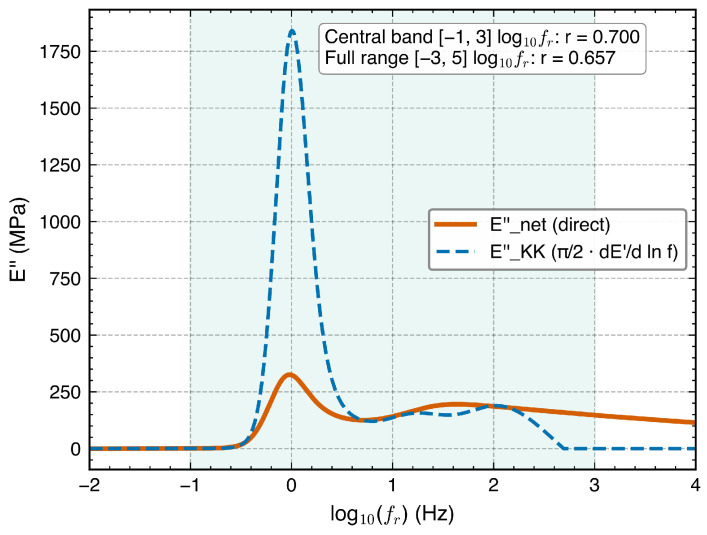
Heuristic Kramers–Kronig consistency check of NeuralWLF predictions at $T_{\mathrm{ref}}=115.8\;{}^{\circ}C$. Network-predicted $E_{\mathrm{net}}^{\prime \prime }$ (solid orange) compared with the approximate K-K derivative $E_{\mathrm{KK}}^{\prime \prime }=\left(\pi /2\right)\;dE^{\prime }/d\left(\ln f\right)$ (dashed blue). Shaded band: central evaluation region ($-1\leq \log_{10}f_{r}\leq 3$). Pearson correlation coefficients: central band r=0.826, full range [−3,5] in $\log_{10}f_{r}$, $r_{\mathrm{full}}=0.786$. The derivative approximation overestimates the peak $E^{\prime \prime }$ by ∼3×, reflecting the known limitations of the local derivative method for narrow loss peaks. This check assesses qualitative covariation not rigorous thermodynamic compliance; integral-form K-K validation would require broader bandwidth data.

Figure 12 shows an approximate Kramers–Kronig plausibility check for the full training model. The local derivative $dE^{\prime }/d\left(\ln f_{r}\right)$ was evaluated by central finite differences on a uniform $\log_{10}f_{r}$ grid (step size $\Delta \log_{10}f_{r}=0.05$), with a Savitzky–Golay smoothing filter (window = 11 points, polynomial order = 3) applied to suppress high-frequency numerical noise. The Pearson correlation was computed over the central band [−1,3] in $\log_{10}f_{r}$, which spans the steepest portion of the $E^{\prime }$ master curve and thus provides the most informative derivative signal; at the boundaries ($\log_{10}f_{r}<-1$ or >3), the derivative approaches zero in the plateau regions, and endpoint truncation artifacts from the finite-difference stencil inflate the numerical noise. The central-band correlation is r=0.826; over the full evaluated range [−3,5], $r_{\mathrm{full}}=0.786$. This indicates that $E^{\prime }$ and $E^{\prime \prime }$ predictions share qualitatively correct covariation; however, the derivative approximation overestimates the $E^{\prime \prime }$ peak by approximately 3×, consistent with the known tendency of the local derivative method to overshoot narrow loss peaks [5]. The K-K check provides moderate evidence of internal consistency within the limited bandwidth but should not be interpreted as rigorous thermodynamic validation, which would require master-curve data spanning multiple decades of frequency. The learned WLF parameters ($C_{1}=12.29$, $C_{2}=51.59\;{}^{\circ}C$) are physically reasonable, with $C_{2}$ close to the universal value of 51.6 °C [12].

### 3.9. Validation Stringency Analysis and Physics-Data Crossover

**Figure 13:**
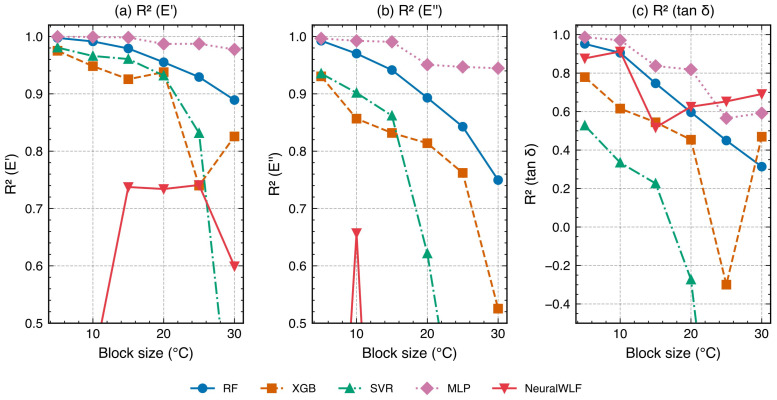
Block size sweep results: $R^{2}$ for (**a**) $\log_{10}E^{\prime }$, (**b**) $\log_{10}E^{\prime \prime }$, and (**c**) tanδ as functions of block size (5–30 °C) for all five models. The tanδ panel reveals the validation stringency gradient: data-driven models (MLP, RF) degrade monotonically with increasing block size, while NeuralWLF maintains more stable performance at large gaps.

To investigate the sensitivity of model evaluation to validation design, a systematic block size sweep was conducted using temperature blocks of 5, 10, 15, 20, 25, and 30 °C (Table 8, Figure 13). This experiment reveals how the ratio of the validation gap to the characteristic feature width—specifically, the FWHM of the tanδ peak (∼10 °C at 1 Hz)—determines the stringency of the validation and the relative performance of data-driven versus physics-informed models.

**Figure 14:**
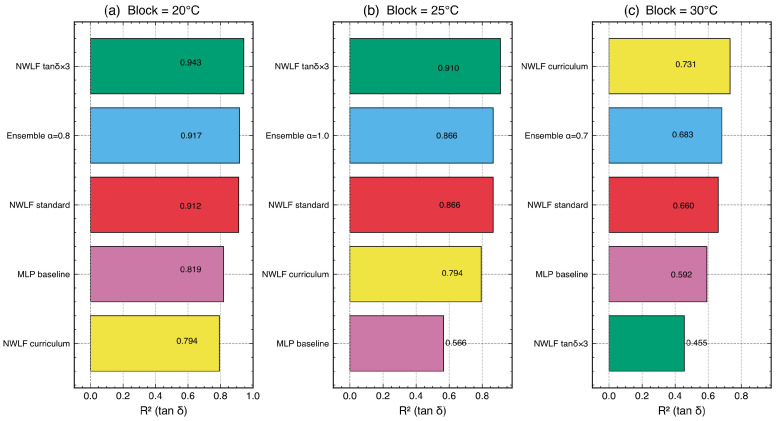
Deep exploration of NeuralWLF training strategies at 20, 25, and 30 °C block sizes: (**a**) 20 °C block, (**b**) 25 °C block, and (**c**) 30 °C block. Each panel compares tanδ$R^{2}$ across configurations (standard joint training, tanδ-weighted loss with λ=3, curriculum learning with WLF frozen for 300 epochs, and MLP+NeuralWLF ensembles).

Validation inflation. At small block sizes (5 °C, gap/FWHM ≈0.5), MLP achieved tanδ$R^{2}=0.986$, suggesting near-perfect prediction. However, as the block size increased to 30 °C (gap/FWHM ≈3.1), MLP performance dropped to $R^{2}=0.592$—a decline of 0.394 in absolute $R^{2}$. This dramatic sensitivity to block size exposes a validation inflation effect: when the gap between training and test data is smaller than the tanδ peak width, adjacent training blocks provide sufficient information for the model to interpolate across the gap without genuinely reconstructing the transition behavior. Conventional random *k*-fold CV, which does not create spatial gaps at all, would yield even more inflated metrics. Detailed architecture-level comparison at strict block settings is shown in Figure 14.

**Figure 15:**
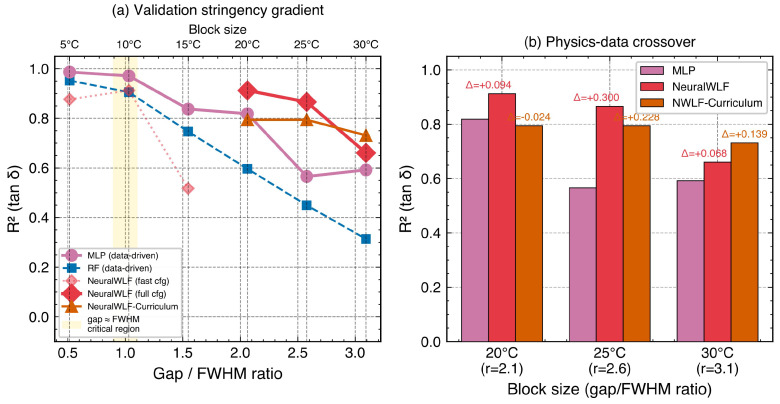
Physics–data crossover analysis. (**a**) tanδ$R^{2}$ versus gap/FWHM ratio for MLP, RF, and NeuralWLF variants; the yellow band marks gap/FWHM =1 (gap equals tanδ peak width). (**b**) Direct comparison of MLP, NeuralWLF (standard), and NeuralWLF (curriculum) at 20, 25, and 30 °C blocks, with Δ values indicating NeuralWLF advantage over MLP.

Physics–data crossover. The block size sweep reveals a crossover phenomenon (Figure 15a): below a critical gap/FWHM ratio of approximately 2.0, MLP (the best data-driven model) outperforms NeuralWLF in tanδ prediction; above this threshold, NeuralWLF becomes superior. At 20 °C blocks (gap/FWHM =2.1), NeuralWLF achieved tanδ$R^{2}=0.912$ versus MLP’s 0.819 (+0.093). At 25 °C blocks (gap/FWHM =2.6), the gap widened to 0.866 versus 0.566 (+0.300)—NeuralWLF’s largest advantage. This crossover has a clear physical interpretation: the WLF layer encodes the constraint that the tanδ peak shape must conform to a time–temperature superposition structure. When the validation gap is large enough that the test block contains the peak maximum while the training data do not, this structural constraint becomes the only available information for reconstructing the transition. Data-driven models, lacking such prior knowledge, cannot extrapolate the peak shape from monotonic regions alone.

Training strategy effects and architecture sensitivity. Deep exploration of NeuralWLF variants (Table 9, Figure 14) revealed clear architecture and objective sensitivity at the most stringent validation level (30 °C blocks). Curriculum learning—freezing the WLF layer for the first 300 epochs and then enabling joint optimization—achieved the highest tanδ$R^{2}$ of 0.731. Standard joint training reached 0.660, while tanδ-weighted loss ($\lambda_{\tan\delta }=3$) dropped to 0.455. This ordering indicates that parameter-regularization strategy is more influential than simply increasing the damping-loss weight under large train–test gaps.

The weighted-loss failure is instructive. Overemphasizing tanδ fitting drove $C_{1}$ and $C_{2}$ to adapt too strongly to the local peak shape in training blocks, which reduced transferability when the held-out block contained a different part of the transition region. Conversely, the curriculum schedule stabilized early feature learning and preserved cross-gap generalization. These results support the view that physics-informed models require balanced optimization of data fit and physically interpretable parameter stability.

Target-dependent model requirements. Ensemble experiments combining MLP predictions for $E^{\prime }$/$E^{\prime \prime }$ with NeuralWLF predictions for tanδ (blending weight α=0.7) demonstrated that the optimal model architecture depends on the target property (Table 9). At 30 °C blocks, the ensemble achieved $E^{\prime }$$R^{2}=0.977$, $E^{\prime \prime }$$R^{2}=0.945$, and tanδ$R^{2}=0.683$—combining the strengths of both approaches. The storage modulus $E^{\prime }$, which exhibits a smooth monotonic decrease through the transition, is well-captured by data-driven models without physics constraints. In contrast, $\tan\delta =E^{\prime \prime }/E^{\prime }$ involves a sharp peak arising from the ratio of two rapidly changing quantities, making it inherently more sensitive to the physical structure of the relaxation process.

## 4. Conclusions

An integrated experimental–computational framework combining multi-frequency DMA characterization with hierarchical ML validation was applied to a commercial PC/ABS blend. The principal findings are as follows:

1. DMA characterization and engineering parameters. Multi-frequency DMA (1–10 Hz) yielded a glass transition range of 115.8–123.2 °C ($E^{\prime \prime }$ peak), a frequency sensitivity of 7.18 °C/decade, and an apparent activation energy of 335±85kJ/, consistent with PC-dominated relaxation. A six-term Prony series ($R^{2}=0.998$) provides FEM-ready constitutive parameters at $T_{\mathrm{ref}}=115.8\;{}^{\circ}C$.
2. Interpolation–extrapolation tradeoff. Temperature-blocked CV ranked MLP as the best interpolator ($\bar{R^{2}}=0.989$), while LOFO validation revealed that the physics-informed NeuralWLF achieved the best cross-frequency generalization ($R^{2}>0.92$ at all held-out frequencies, including the 1 Hz edge where MLP collapsed to $R^{2}=-0.97$). Among data-driven models, RF provided the best interpolation–extrapolation balance.
3. Validation inflation and the gap/FWHM criterion. A block size sweep (5–30 °C) exposed a validation inflation effect: MLP tanδ$R^{2}$ dropped from 0.986 to 0.592 as the gap-to-FWHM ratio increased from 0.5 to 3.1. Honest evaluation of DMA–ML models requires validation gaps comparable to or exceeding the characteristic feature width (tanδ FWHM).
4. Physics–data crossover. A crossover at gap/FWHM ≈2 separates a data-driven regime (where MLP suffices) from a physics-informed regime (where NeuralWLF becomes essential). At gap/FWHM =2.6, NeuralWLF outperformed MLP by +0.300 in tanδ$R^{2}$; curriculum learning (freezing the WLF layer for 300 epochs) further improved the most stringent validation ($R^{2}=0.660\to 0.731$), demonstrating that physics-parameter regularization outweighs loss weighting for extrapolation.
5. Target-dependent model selection. $E^{\prime }$ and $E^{\prime \prime }$ (smooth, monotonic) are well-captured by data-driven MLP ($R^{2}>0.97$), whereas tanδ (sharp peak, ratio quantity) benefits from physics-informed constraints. The gap/FWHM ratio and target property together provide a quantitative criterion for selecting between data-driven and physics-informed architectures, generalizable to other polymer systems and relaxation-dominated property predictions.

Scope statement. This work is positioned as a methodological DMA–ML validation study on a single commercial PC/ABS system rather than as a fundamental advancement of polymer viscoelastic theory.

Limitations. The present study is constrained by (i) a single-decade frequency range limiting the precision of activation energy estimation and TTS validation, (ii) incomplete material characterization (no DSC, TGA, or compositional analysis), and (iii) single-specimen testing precluding assessment of batch-to-batch variability.

Future work should extend this framework to broader frequency ranges (0.01–100 Hz), explore domain adaptation for cross-material transfer, and investigate whether the gap/FWHM crossover criterion applies to other relaxation phenomena beyond glass transitions.

## Figures and Tables

**Table 1:** Neural-network configuration and training parameters used in this study.

Item	Setting
MLP baseline (data-driven)	Hidden layers (128, 64, 32), ReLU activation, Adam optimizer, learning rate 1 × 10^−3^, max iterations 2000, early stopping enabled (validation fraction 0.15), random seed 42.
NeuralWLF inputs	Temperature *T*, $\log_{10}f$, and learned reduced-frequency feature $\log_{10}f_{r}=\log_{10}f+\log_{10}a_{T}$.
NeuralWLF backbone	Width 128, three hidden transforms with GELU activation (num_layers = 4 implementation setting).
Output heads	Independent heads for $\log_{10}E^{\prime }$, $\log_{10}E^{\prime \prime }$, and tanδ; Softplus activation on tanδ head.
CV/LOFO optimization	Adam with two parameter groups: neural 5 × 10^−3^, WLF 1 × 10^−3^; warmup 50 epochs; ReduceLROnPlateau (patience 100, factor 0.5); early stopping patience 600.
CV/LOFO losses	MSE losses for $\log_{10}E^{\prime }$, $\log_{10}E^{\prime \prime }$, and tanδ (weights 1:1:1), WLF regularization $\lambda_{\mathrm{WLF}}=5\;\times 10^{-4}$, *T*-jitter augmentation (σ=0.5 °C).
Full-physics run (K-K plausibility)	Adds approximate K-K penalty ($\lambda_{\mathrm{KK}}=0.02$), monotonicity penalty ($\lambda_{\mathrm{mono}}=0.05$), and curriculum schedule with WLF frozen for first 300 epochs.

**Table 2:** Summary of DMA parameters from single cantilever multi-frequency temperature sweeps ^a^.

Freq. (Hz)	$E_{\mathrm{gl}.}^{\prime }$ (MPa)	$E_{\mathrm{rub}.}^{\prime }$ (MPa)	$T_{g,\tan\delta }$ (°C)	tanδ pk.	$T_{g,E^{\prime \prime }}$ (°C)	$E^{\prime \prime }$ pk. (MPa)	FWHM (°C)	Status
1.0	1871	5.5	123.5	2.36	115.8	330	9.7	Used
2.0	1777	5.6	128.0	2.19	120.9	337	9.9	Used
5.0	1863	7.1	130.1	1.90	123.2	337	11.5	Used
10.0	1810	9.6	130.4	1.70	123.2	329	12.2	Used

^a^ The 0.5 Hz data contained only 94 data points (versus ∼930 at other frequencies), resulting in anomalous tanδ peak and narrow FWHM. This row is shown for completeness but is excluded from all quantitative analyses, including frequency regressions, TTS construction, ML training, and trend discussions. See Section 3.2.

**Table 3:** Frequency dependence parameters of the glass transition.

Parameter	tanδ Method	$E^{\prime \prime }$ Method
$dT_{g}/d\left(\log f\right)$ (°C/decade)	6.69	7.18
$E_{a}$ ($kJ\;{\mathrm{mol}}^{-1}$)	—	335
$T_{\mathrm{ref}}$ (°C)	—	115.8

**Table 4:** Activation energy and TTS parameters with literature comparison.

Material	$dT_{g}/d\left(\log f\right)$ (°C/Decade)	$E_{a}$ (kJ mol^−1^)	$T_{\mathrm{ref}}$ (°C)
This work (PC/ABS)	7.18	335	115.8
PC/ABS (literature)	3–7	200–400	$T_{g}$
Pure PC	5–6	300–400	150

**Table 5:** Six-term Prony series parameters ($T_{\mathrm{ref}}=115.8\;{}^{\circ}C$, $R^{2}=0.998$).

Term	Modulus $E_{i}$ (MPa)	Relaxation Time $\tau_{i}$ (s)
$E_{\infty }$	5.4	—
$E_{1}$ ^b^	0.25	$6.04\times 10^{6}$
$E_{2}$ ^b^	5.1	3777
$E_{3}$	385	0.450
$E_{4}$	478	0.065
$E_{5}$	390	0.065
$E_{6}$ ^b^	464	$1.87\times 10^{-3}$

^b^ Weakly identifiable terms whose relaxation times fall outside the observable experimental window. For FEM implementation, these parameters should be used only within $f_{r}\approx 10^{-2}$–$10^{3}$ Hz at $T_{\mathrm{ref}}$; extrapolation beyond this range inherits TTS imperfections of the immiscible blend and the non-identifiability of boundary terms.

**Table 6:** Temperature-blocked cross-validation results for ML prediction of viscoelastic properties ^a^.

	$\log_{10}E^{\prime }$		$\log_{10}E^{\prime \prime }$		tanδ		
Model	$R^{2}$	RMSE	$R^{2}$	RMSE	$R^{2}$	RMSE	$\bar{R^{2}}$
*Panel A: All 14 temperature blocks (full range, 30–170 °C)*							
MLP	0.997	0.054	0.991	0.060	0.978	0.064	0.989
RF	0.991	0.096	0.971	0.111	0.889	0.143	0.950
XGB	0.948	0.229	0.857	0.245	0.624	0.264	0.810
SVR	0.966	0.186	0.902	0.203	0.324	0.354	0.731
*Panel B: Transition-zone blocks only (90–150 °C, 6 blocks) ^b^*							
MLP	0.992	0.048	0.985	0.055	0.971	0.058	0.983
RF	0.982	0.072	0.958	0.092	0.860	0.127	0.933
NeuralWLF ^c^	0.998	0.029	0.998	0.040	0.985	0.065	0.994

^a^ Panel A reports data-driven models over all 14 blocks (full temperature range). Panel B reports transition-zone blocks where target variance is sufficient for meaningful $R^{2}$. ^b^ Blocks restricted to 90–150 °C to exclude glassy and rubbery plateaus where ${\mathrm{SS}}_{\mathrm{tot}}\to 0$ renders $R^{2}$ undefined. MLP and RF values in Panel B are recomputed over these 6 blocks only; XGB and SVR omitted for brevity (ranking unchanged). ^c^ Physics-informed model with embedded WLF layer. Full-range (all 14 blocks) MAE: 0.21/0.04/0.11 for $\log_{10}E^{\prime }$/$\log_{10}E^{\prime \prime }$/tanδ, higher than MLP (0.03/0.04/0.04), reflecting limited advantage in thermally flat plateau regions.

**Table 7:** Leave-one-frequency-out (LOFO) validation results for all four data-driven models and NeuralWLF.

Held-Out Freq. (Hz)	Model	$R^{2}$($\log_{10}E^{\prime }$)	$R^{2}$($\log_{10}E^{\prime \prime }$)	$R^{2}$(tanδ)
*Panel A: Data-driven models*				
1 (edge)	RF	0.961	0.894	0.553
1 (edge)	XGB	0.939	0.789	0.433
1 (edge)	SVR	−1.184	−5.797	0.254
1 (edge)	MLP	0.911	0.294	−0.972
2	RF	0.957	0.900	0.488
2	XGB	0.990	0.980	0.923
2	SVR	0.982	0.906	0.580
2	MLP	0.986	0.966	0.904
5 (interior)	RF	0.996	0.970	0.985
5 (interior)	XGB	0.979	0.946	0.811
5 (interior)	SVR	0.989	0.950	0.642
5 (interior)	MLP	0.984	0.965	0.969
10 (edge)	RF	0.995	0.977	0.984
10 (edge)	XGB	0.973	0.919	0.803
10 (edge)	SVR	−0.702	−5.135	−1.327
10 (edge)	MLP	0.857	0.807	0.380
*Panel B: Physics-informed model*				
1 (edge)	NeuralWLF	0.992	0.980	0.922
2	NeuralWLF	0.999	0.995	0.987
5 (interior)	NeuralWLF	0.999	0.990	0.997
10 (edge)	NeuralWLF	0.999	0.963	0.989

**Table 8:** Block size sweep results: $R^{2}$ for all five models at block sizes of 5–30 °C ^a^.

Block (°C)	Gap/FWHM	Model	$R^{2}$($\log_{10}E^{\prime }$)	$R^{2}$($\log_{10}E^{\prime \prime }$)	$R^{2}$(tanδ)
5	0.5	MLP	0.999	0.997	0.986
		RF	0.997	0.988	0.960
10	1.0	MLP	0.997	0.991	0.978
		RF	0.991	0.971	0.889
15	1.5	MLP	0.993	0.978	0.924
		RF	0.973	0.920	0.721
20	2.1	MLP	0.986	0.960	0.819
		NWLF	0.871	0.818	0.912
25	2.6	MLP	0.983	0.955	0.566
		NWLF	0.871	0.818	0.866
30	3.1	MLP	0.977	0.945	0.592
		NWLF	0.871	0.818	0.660

^a^ FWHM of tanδ peak =9.7 °C at 1 Hz. NWLF = NeuralWLF (standard joint training). Only MLP (best data-driven) and NeuralWLF shown for clarity; full data available in the data repository (see Data Availability).

**Table 9:** Deep exploration of NeuralWLF training strategies at 20, 25, and 30 °C block sizes.

Configuration	Block (°C)	$R^{2}$($\log_{10}E^{\prime }$)	$R^{2}$($\log_{10}E^{\prime \prime }$)	$R^{2}$(tanδ)
MLP baseline	30	0.977	0.945	0.592
NWLF standard	30	0.871	0.818	0.660
NWLF tanδ-weighted (λ=3)	30	0.871	0.818	0.455
NWLF curriculum	30	0.871	0.818	0.731
Ensemble (α=0.7)	30	0.977	0.945	0.683
MLP baseline	25	0.983	0.955	0.566
NWLF standard	25	0.871	0.818	0.866
NWLF curriculum	25	0.871	0.818	0.794
MLP baseline	20	0.986	0.960	0.819
NWLF standard	20	0.871	0.818	0.912
NWLF curriculum	20	0.871	0.818	0.794

## Data Availability

The processed DMA dataset (temperature–frequency–property CSV files for all five single-cantilever frequencies and three dual-cantilever specimens), ML training scripts with fixed random seeds (RANDOM_STATE = 42) and hyperparameters, temperature-blocked CV partition definitions (14 blocks × 10 °C), LOFO fold indices, and a Jupyter notebook reproducing all figures and tables from the processed CSV data will be deposited in a public repository (Zenodo, with DOI) upon acceptance of this manuscript. During peer review, a private, anonymous-access Zenodo record containing the complete reproducibility package (processed data, analysis code, and figure-reproduction notebook) will be made available to the editor and reviewers; the access link will be provided in the cover letter or upon editorial request. The raw instrument files (.xls) are available from the corresponding author upon reasonable request.

## Abbreviations

The following abbreviations are used in this manuscript:

DMA	Dynamic Mechanical Analysis
TTS	Time–Temperature Superposition
WLF	Williams–Landel–Ferry
FEM	Finite Element Method
ML	Machine Learning
RF	Random Forest
XGB	Extreme Gradient Boosting
SVR	Support Vector Regression
MLP	Multilayer Perceptron
CV	Cross-Validation
LOFO	Leave-One-Frequency-Out
FWHM	Full Width at Half Maximum
K-K	Kramers–Kronig

## References

[1] Balart R., López J., García D., Dolores Salvador M. (2005). Recycling of ABS and PC from electrical and electronic waste. Effect of miscibility and previous degradation on final performance of industrial blends. Eur. Polym. J..

[2] Seo J.S., Jeon H.T., Han T.H. (2020). Rheological Investigation of Relaxation Behavior of Polycarbonate/Acrylonitrile-Butadiene-Styrene Blends. Polymers.

[3] Dawoud M., Taha I., Ebeid S.J. (2016). Mechanical behaviour of ABS: An experimental study using FDM and injection moulding techniques. J. Manuf. Process..

[4] Sood A.K., Ohdar R.K., Mahapatra S.S. (2010). Parametric appraisal of mechanical property of fused deposition modelling processed parts. Mater. Des..

[5] Ferry J.D. (1980). Viscoelastic Properties of Polymers.

[6] Menard K.P., Menard N.R. (2020). Dynamic Mechanical Analysis.

[7] McCrum N.G., Read B.E., Williams G. (1967). Anelastic and Dielectric Effects in Polymeric Solids.

[8] Liu X., Tian J., Li X., Chen J., Li J. (2023). Temperature and Frequency Dependence of the Dynamic Viscoelastic Properties of Silicone Rubber. Polymers.

[9] (2018). Standard Test Method for Assignment of the Glass Transition Temperature by Dynamic Mechanical Analysis.

[10] David D.J., Sincock T.F. (1992). Estimation of miscibility of polymer blends using the solubility parameter concept. Polymer.

[11] Chiang W.Y., Hwung D.S. (1987). Properties of polycarbonate/acrylonitrile-butadiene-styrene blends. Polym. Eng. Sci..

[12] Williams M.L., Landel R.F., Ferry J.D. (1955). The Temperature Dependence of Relaxation Mechanisms in Amorphous Polymers and Other Glass-forming Liquids. J. Am. Chem. Soc..

[13] Angell C.A. (1991). Relaxation in liquids, polymers and plastic crystals—strong/fragile patterns and problems. J. Non-Cryst. Solids.

[14] Shangguan Y., Chen F., Jia E., Lin Y., Hu J., Zheng Q. (2017). New Insight into Time-Temperature Correlation for Polymer Relaxations Ranging from Secondary Relaxation to Terminal Flow: Application of a Universal and Developed WLF Equation. Polymers.

[15] Colby R.H. (1989). Breakdown of time-temperature superposition in miscible polymer blends. Polymer.

[16] Roland C.M., Ngai K.L. (1992). Segmental relaxation and the correlation of time and temperature dependences in poly(vinyl methyl ether)/polystyrene mixtures. J. Rheol..

[17] Genovese A., Farroni F., Sakhnevych A. (2022). Fractional Calculus Approach to Reproduce Material Viscoelastic Behavior, including the Time-Temperature Superposition Phenomenon. Polymers.

[18] Park S.W., Schapery R.A. (1999). Methods of interconversion between linear viscoelastic material functions. Part I—a numerical method based on Prony series. Int. J. Solids Struct..

[19] Emri I., Tschoegl N.W. (1993). Generating line spectra from experimental responses. Part I: Relaxation modulus and creep compliance. Rheol. Acta.

[20] Neubauer M., Pohl M., Kuber M., Glück N., Wiedemann M., Kowalski J. (2022). DMA of TPU Films and the Modelling of Their Viscoelastic Properties for Noise Reduction in Jet Engines. Polymers.

[21] Chen L., Pilania G., Batra R., Huan T.D., Kim C., Kuenneth C., Ramprasad R. (2021). Polymer informatics: Current status and critical next steps. Mater. Sci. Eng. R Rep..

[22] Liu Y., Zhao T., Ju W., Shi S. (2017). Materials discovery and design using machine learning. J. Mater..

[23] Pilania G., Iverson C.N., Lookman T., Marrone B.L. (2019). Machine-learning-based predictive modeling of glass transition temperatures: A case of polyhydroxyalkanoate homopolymers and copolymers. J. Chem. Inf. Model..

[24] Gu G.X., Chen C.T., Buehler M.J. (2018). De novo composite design based on machine learning algorithm. Extrem. Mech. Lett..

[25] Mannodi-Kanakkithodi A., Pilania G., Huan T.D., Lookman T., Ramprasad R. (2016). Machine learning strategy for accelerated design of polymer dielectrics. Sci. Rep..

[26] Jha D., Ward L., Paul A., Liao W.k., Choudhary A., Wolverton C., Agrawal A. (2018). ElemNet: Deep learning the chemistry of materials from only elemental composition. Sci. Rep..

[27] Merchant A., Batzner S., Schoenholz S.S., Aykol M., Cheon G., Cubuk E.D. (2023). Scaling deep learning for materials discovery. Nature.

[28] Qin B., Zhong Z. (2024). A Physics-Guided Machine Learning Model for Predicting Viscoelasticity of Solids at Large Deformation. Polymers.

[29] Jain A., Gurnani R., Rajan A., Qi H.J., Ramprasad R. (2025). A physics-enforced neural network to predict polymer melt viscosity. npj Comput. Mater..

[30] Malashin I., Tynchenko V., Gantimurov A., Nelyub V., Borodulin A. (2025). Physics-Informed Neural Networks in Polymers: A Review. Polymers.

[31] Hastie T., Tibshirani R., Friedman J. (2009). The Elements of Statistical Learning.

[32] Chen T., Guestrin C. XGBoost: A Scalable Tree Boosting System. Proceedings of the 22nd ACM SIGKDD International Conference on Knowledge Discovery and Data Mining.

[33] Karniadakis G.E., Kevrekidis I.G., Lu L., Perdikaris P., Wang S., Yang L. (2021). Physics-informed machine learning. Nat. Rev. Phys..

[34] Raissi M., Perdikaris P., Karniadakis G.E. (2019). Physics-informed neural networks: A deep learning framework for solving forward and inverse problems involving nonlinear partial differential equations. J. Comput. Phys..

[35] Strobl C., Boulesteix A.L., Zeileis A., Hothorn T. (2007). Bias in random forest variable importance measures: Illustrations, sources and a solution. BMC Bioinform..

